# A systematic review and meta-analysis of studies comparing muscle-in-vein conduits with autologous nerve grafts for nerve reconstruction

**DOI:** 10.1038/s41598-021-90956-3

**Published:** 2021-06-03

**Authors:** Johannes C. Heinzel, Mai Quyen Nguyen, Laura Kefalianakis, Cosima Prahm, Adrien Daigeler, David Hercher, Jonas Kolbenschlag

**Affiliations:** 1 Department of Hand-, Plastic, Reconstructive and Burn Surgery, BG Klinik Tuebingen, Eberhard Karls University, Schnarrenbergstraße 95, Tuebingen, Germany; 2 Ludwig Boltzmann Institute for Experimental and Clinical Traumatology, Donaueschingenstraße 13, Vienna, Austria; 3 Austrian Cluster for Tissue Regeneration, Vienna, Austria

**Keywords:** Translational research, Trauma, Outcomes research, Neurological disorders

## Abstract

The gold-standard method for reconstruction of segmental nerve defects, the autologous nerve graft, has several drawbacks in terms of tissue availability and donor site morbidity. Therefore, feasible alternatives to autologous nerve grafts are sought. Muscle-in-vein conduits have been proposed as an alternative to autologous nerve grafts almost three decades ago, given the abundance of both tissues throughout the body. Based on the anti-inflammatory effects of veins and the proregenerative environment established by muscle tissue, this approach has been studied in various preclinical and some clinical trials. There is still no comprehensive systematic summary to conclude efficacy and feasibility of muscle-in-vein conduits for reconstruction of segmental nerve defects. Given this lack of a conclusive summary, we performed a meta-analysis to evaluate the potential of muscle-in-vein conduits. This work’s main findings are profound discrepancies regarding the results following nerve repair by means of muscle-in-vein conduits in a preclinical or clinical setting. We identified differences in study methodology, inter-species neurobiology and the limited number of clinical studies to be the main reasons for the still inconclusive results. In conclusion, we advise for large animal studies to elucidate the feasibility of muscle-in-vein conduits for repair of segmental defects of critical size in mixed nerves.

## Introduction

Peripheral nerve injuries, which affect up to 60 out of 1.000.000 people in the US annually are frequently associated with devastating live-long sequelae for the affected patients and a high socioeconomic burden^1,2^. Neurotmesis, e.g. discontinuity of the entire nerve, always requires surgical treatment to reconstruct the severed nerve’s continuity^3^. In cases of segmental nerve damage in which tension-free neurorrhaphy is impossible, autologous nerve grafts (ANGs) are considered the gold-standard treatment^4^. However, their use is restricted due to limited availability throughout the body and specific requirements regarding length, diameter, and vascularization. Additionally, harvesting of a donor nerve results in loss of motor and/or sensory function which must be balanced against the expectable gain of function by means of the reconstructed recipient nerve^5^. Allogeneic nerve transplants can only be used in decellularized form due to the imminent graft-versus-host reaction and are also cost-intensive^6^. Scientists aiming to develop an adequate alternative for ANGs by means of tissue- and bio-engineering have devised and tested a multitude of biologic and synthetic materials to create an ideal conduit to bridge peripheral nerve defects^7^. Use of veins as biological nerve guidance conduits was pioneered by Wrede at the beginning of the twentieth century^8^ and reinvented by Chiu et al. in the 1980s^9^. Although good results were reported for bridging of short defects, the vein’s tendency to collapse over distances exceeding 1–2 cm^10^ demanded a further refinement of the technique. In 1993, Brunelli and colleagues pioneered use of a muscle-in-vein conduit (MVC), combining the vein wall’s shielding and anti-inflammatory capabilities with the proregenerative effects of the muscle tissue^11^. MVC are prepared by harvesting a segment of an autologous vein and some muscle fibers which are then pulled inside the vein using fine forceps or a needle holder, yielding an autologous nerve guidance conduit. The MVC is then interposed between the severed nerve stumps, taking care to pull them 1–2 mm inside the vein to guarantee adequate entubulization^12^. The muscle fibers’ basal membranes act as natural guidance channels for regenerating axons while the muscle also prevents collapsing of the vein^13^. Following promising initial results obtained by Brunelli et al., the new reconstructive approach was further evaluated both in preclinical as well as clinical studies with increasing interest in the late 1990s and beginning of the twenty-first century^10,14,15^. Over the past three decades, multiple authors evaluated use of MVCs to reconstruct peripheral nerve defects in both animals and human patients, reporting divergent results. While some authors conducting clinical studies did report non-inferiority of MVCs compared to ANGs^12,16^ this has been opposed by reports of strong inferiority of MVCs in rodent models of segmental peripheral nerve injury ^17,18^.

To the best of our knowledge, one systematic meta-analysis which compared the effects of MVCs on sensory recovery after digital nerve reconstruction to other reconstructive approaches has been published so far^19^. The outcomes were equivalent among all included reconstructive methods. However, this work was published in 2013 and since then a multitude of preclinical and some clinical studies regarding the use of MVCs has been conducted and published. Given this increment both in reported patient outcomes and results from animal studies, a comprehensive analysis of both sets of data is still pending yet. In the light of the high need for alternatives to reconstruct peripheral nerves by means of grafting material, a comprehensive evaluation of the published data regarding MVCs will provide important insight into the actual feasibility and potential of this approach. This study therefore presents the first systematic review and meta-analysis of studies evaluating MVCs to reconstruct peripheral nerves both in rats as well in human patients. It is the aim of this work to give an overview on the divergent outcomes after nerve repair by this technique and to discuss the potential mechanisms underlying these results. We aim to provide preclinical scientist and clinicians with a broad knowledge base regarding the published data on the use of MVCs for peripheral nerve repair.

## Results

### Results of the systematic literature research

**Figure 1:**
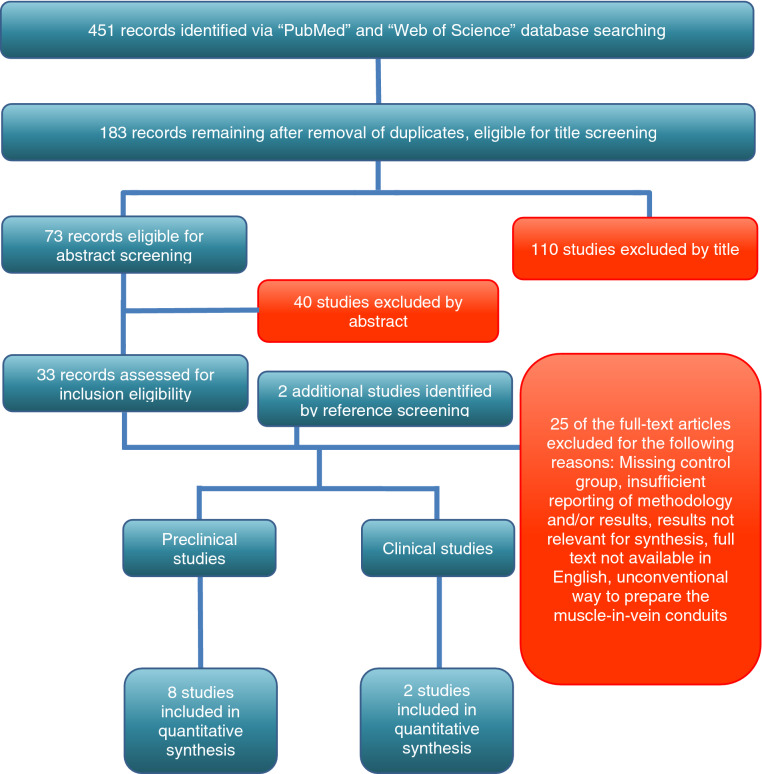
Flow diagram of the search and study selection process. The selection process, based on the PRISMA-guidelines, is depicted in chronological order.

10 studies, 8 preclinical and 2 clinical, met the inclusion criteria and were consecutively included in the quantitative synthesis. A total of 451 studies were identified, of which 183 remained for title and abstract screening after removal of duplicates. In accordance with the inclusion criteria (Table 1), 110 studies were excluded after title screening, whereas 40 studies were excluded after abstract screening. Therefore, 33 studies remained to be assessed for eligibility via full text screening. Two additional studies could be identified via reference checking. Out of these 35 studies, 25 studies were excluded due to various reasons. The detailed selection process is illustrated in Fig. 1.

**Table 1:** Inclusion criteria for the systematic review and meta-analysis.

Preclinical studies	
	*Inclusion criteria*
Population	Peripheral motor nerve lesion in rat
Intervention	Muscle-in-vein conduit (MVC)
Comparison	Autologous nerve graft (ANG)
Outcome	*Voluntary motor function* Walking Track Analysis Grasping Strength *Electrophysiology:* Nerve conduction velocity (NCV) Compound Muscle Action Potential (CMAP) *Histology* Axon count, axon density, axon diameter, fiber diameter, thickness of the myelin sheath,
Study design	Experimental study in the rat Segmental nerve injury Study in English or German language
Clinical studies	
	*Inclusion criteria*
Population	Peripheral nerve lesion in humans
Intervention	Muscle-in-vein conduit (MVC)
Comparison	Autologous nerve graft
Outcome	*Sensory function:* 2-point discrimination
Study design	Segmental nerve injury Study in English or German language

### Characteristics of included studies

#### Preclinical studies

Eight preclinical studies were available in English and used either the sciatic nerve (n = 6) or median nerve (n = 2) injury model. A total of 245 animals was investigated (median nerve: n = 88, sciatic nerve: n = 157). The smallest experimental groups consisted of 3 animals^11,20^ while the largest comprised 14 animals^21^. The mean nerve defect size was 13 mm while the shortest and longest experimental nerve defect measured 7 mm^18^ and 20 mm^11,22^ respectively. The mean observation period was 20 weeks or 143 days, respectively. While several authors chose to observe the animals for 12 weeks, the longest observational period was 10 months^21^.

#### Clinical studies

The two clinical studies included were conducted in a whole of 46 patients with either median or ulnar nerve^16^ or digital nerve injuries^12^. In total, 49 nerve defects were reconstructed with either ANGs (n = 26) or MVCs (n = 23). Defect lengths ranged from 10 to 60 mm. While the ANGs were harvested from the medial antebrachial cutaneous nerve^12^ or sural nerve^16^, the origins of the MVCs were not further specified. In the one study the patients’ age ranged from 11 to 72 years and patients were observed between 12 and 58 months^12^. The other study’s authors reported a mean age of around 27 years, but the observation period was not specified^16^. Both studies assessed static two-point discrimination as common outcome measure, while dynamic two-point discrimination, Semmes–Weinstein monofilaments, and muscle power according to the Medical Research Council Scale were also evaluated in either the one or the other study.

### Effects of interventions

#### Preclinical studies

*Functional evaluation*

*Sciatic nerve model*

Out of the eight included studies, five reported outcome metrics of functional recovery after nerve reconstruction via either MVC or ANG. Walking track analysis was used in two studies which featured the sciatic nerve model and a total of 45 rats. MVC treated animals displayed either significantly worse (p < 0.01) motor recovery than those treated with ANGs^22^ or had to be excluded after 4 weeks of follow-up due to severe limb autotomy^20^. Ramli et al. assessed functional recovery also by means of the Pinch test. Although the authors did not report any statistical analysis of this data, the published data sheet indicated no marked differences between the ANG and MVC group^20^.

*Median nerve model*

In the three studies evaluating MVCs versus ANGs in the rat median nerve model, motor function recovery was assessed in a total of 108 rodents by means of the grasping test. One study^18^ with 16 rats featured the staircase test^23^ as an additional assessment tool. Regarding the grasping test, in one study rats treated with MVCs recovered significantly (p < 0.05) less grasping strength than rats treated with ANGs at six months postoperatively^21^, while another study reported no significant differences between the two groups^24^. Stossel et al. did not report absolute grasping strength in grams but made use of a trinary scoring system to evaluate the rats’ grasping ability. While they found no significant differences between the two groups at postoperative week (WPO) 4 and WPO12, significantly (p < 0.05) more animals treated with ANGs had regained the ability to pull the bar of the testing apparatus with measurable force in comparison to the MVC group at WPO8. In regard to the staircase test, the same authors reported a significant difference (p < 0.05) in favor of the ANG group at WPO4 while there were no significant differences detectable at WPO8 and WPO12^18^.

*Electrophysiological evaluations*

Electrophysiological evaluations including nerve conduction velocity and the amplitude of the evoked compound muscle action potential (CMAP) were performed in 3 studies and a total of 87 rats.

*Median nerve model*

One study^18^ assessed the CMAP amplitudes of the thenar muscles by means of minimally invasive measurements in 16 rats every four weeks following median nerve resection and repair. At WPO4 and WPO8, respectively, no significant differences were detectable between the ANG and MVC group whereas CMAP amplitudes were highly significantly (p < 0.01) larger in rats treated with ANGs at WPO12. In addition, CMAP amplitudes in the ANG groups were highly significantly (p < 0.01) increased at WPO12 compared to WPO8. However, this difference was not significant in the MVC group.

*Sciatic nerve model*

Two studies^17,20^ evaluated electrophysiological outcome metrics after sciatic nerve injury and repair in a total of 71 rats. Stossel et al. found that CMAP amplitudes recorded from the tibialis anterior muscles of MVC-treated animals were highly significantly (p < 0.001) lower than those of the ANG group at DPO60, DPO90 and DPO120. While CMAP amplitudes in the ANG group were highly significantly higher at DPO90 (p < 0.001) and DPO120 (p < 0.01) in comparison to DPO60. This difference was not significant in the MVC group^17^. The authors also calculated the estimated axon loss based on their electrophysiologic recordings and found significant (p < 0.05) lower numbers of axons in the MVC group compared to the ANG group at DPO60. This difference was highly significant (p < 0.01) at DPO90 and DPO120, respectively. Additionally, Stossel et al. also performed the same measurements on the plantaris longus muscle. While they found no significant group differences at DPO60, animals of the ANG group had significantly (p < 0.05) larger CMAP amplitudes at DPO90. This difference was highly significant (p < 0.001) at DPO120. While there was no significant recovery of CMAP amplitudes in the MVC group at DPO120 compared to earlier time points, this recovery was highly significant compared to DPO60 (p < 0.001) and DPO90 (p < 0.01) in the ANG group. In regard to the estimated axonal loss, this was highly significantly more pronounced (p < 0.001) in the MVC group compared to the ANG group at DPO90 and DPO120. While a highly significant (p < 0.001) increment in axon numbers compared to baseline was observable in the ANG group at DPO90 and DPO120, this did not occur in the MVC group.

Ramli and colleagues did not detect significant differences regarding CMAP amplitude, CMAP onset latency and nerve conduction velocity between the MVC and ANG group^20^.

*Histological evaluations*

Seven of the eight included preclinical studies (median nerve = 2, sciatic nerve = 5) with a total of 173 rats featured histological analysis of the reconstructed nerve, including 1) number of axons, 2) diameter of axons, 3) mean density as well as 4) size of myelinated fibers and 5) thickness of the myelin sheath.

*Median nerve model*

Papalia et al. found no significant differences regarding the total number, fiber diameter or myelin thickness between the ANG and MVC group at 6 months postoperatively^24^, whereas Stossel’s group reported significantly (p < 0.05) and highly significantly (p < 0.001) lower numbers of myelinated axons in the MVC group at WPO8 and WPO12, respectively. The same authors observed no significant differences regarding the repaired nerves’ mean cross sectional area, axon diameter, fiber diameter, g-ratio or myelin thickness between groups, but found a highly significantly (p < 0.001) increased nerve fiber density in the ANG group compared to the MVC group at WPO12^18^.

*Sciatic nerve model*

Brunelli and colleagues, who bridged 10 mm and 20 mm nerve defects of the sciatic nerve in their study, found a significantly increased number of axons in the distal nerve stumps of animals treated with MVCs when compared to the ANG group in both settings^11^.

Geuna et al. assessed the total number, mean size and fiber density of myelinated nerve fibers and found no significant differences between the two groups^25^.

Another study which evaluated histological parameters at DPO120 found that the total number of fibers was significantly (p < 0.05) lower in the MVC group compared to the ANG group whereas there were no significant differences regarding nerve fiber density, axon diameter, fiber diameter, g-ratio and myelin thickness ^17^.

Ramli and colleagues assessed the (1) number of nerve fibers, (2) degree of angiogenesis, (3) infiltration of immune cells, (4) infiltration of muscle cells and (5) development of traumatic neuroma. These authors did not report results of any statistical comparison between groups but found a higher number of nerve fibers within the distal stumps of nerves reconstructed with an ANG. While the degree of angiogenesis, immune cell infiltration and muscle cell infiltration was equivalent between both groups, abundant neuroma formation was apparent in nerves repaired with MVCs. No neuroma formation was observable in the ANG group^20^.

Ulkur et al. evaluated the number of myelinated axons and the mean axonal diameter at WPO28, reporting significantly (p < 0.05) higher numbers in regard to both counts in the ANG group^22^.

*Muscle weight*

Stossel’s group assessed muscle weight of both the tibialis anterior muscle and gastrocnemius muscle at DPO120 following sciatic nerve repair, which was significantly increased in the ANG group compared to the MVC group^17^.

#### Clinical studies

Manoli’s group found no statistically significant differences in regard to static or moving two-point discrimination as well as the Semmes–Weinstein-Monofilament test after digital nerve repair (n = 31) with either an ANG (n = 14) or a MVC (n = 17)^12^. Ahmad et al. also found no statistically significant differences in regard to two-point discrimination or muscle power assessed by means of the Medical Research Council scale following nerve repair (n = 18) with either ANGs (n = 9) or MVCs (n = 9)^16^.

### Meta-analysis

**Figure 2:**
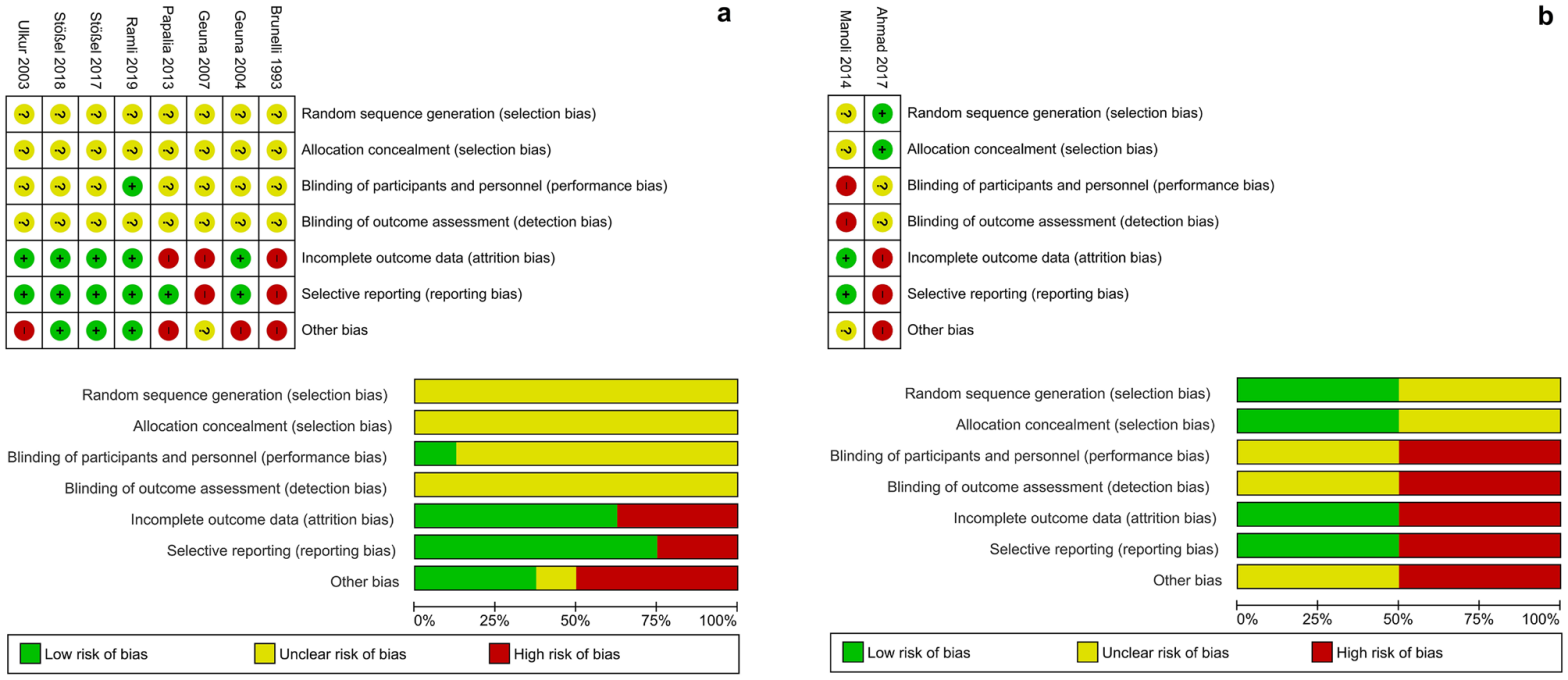
Assessed risk of bias of all preclinical (**a**) and clinical (**b**) studies included in the meta-analysis. As recommended by the PRISMA guidelines, the authors performed an analysis of bias, which could result in over- or underestimation of the effect of interventions, in all included studies. Depicted are the scored risk of bias for individual items (upper part) and the percentage of the assessed bias risk (bottom part) across all preclinical (**a**) and clinical (**b**) studies included in the meta-analysis.

Our meta-analysis revealed marked differences regarding the outcome of nerve repair by means of ANGs or MVCs between preclinical and clinical studies. Figure 2 depicts the quality of all included preclinical studies (Fig. 2a) and clinical studies (Fig. 2b) in regard to the risk of bias.

#### Preclinical studies

**Figure 3:**
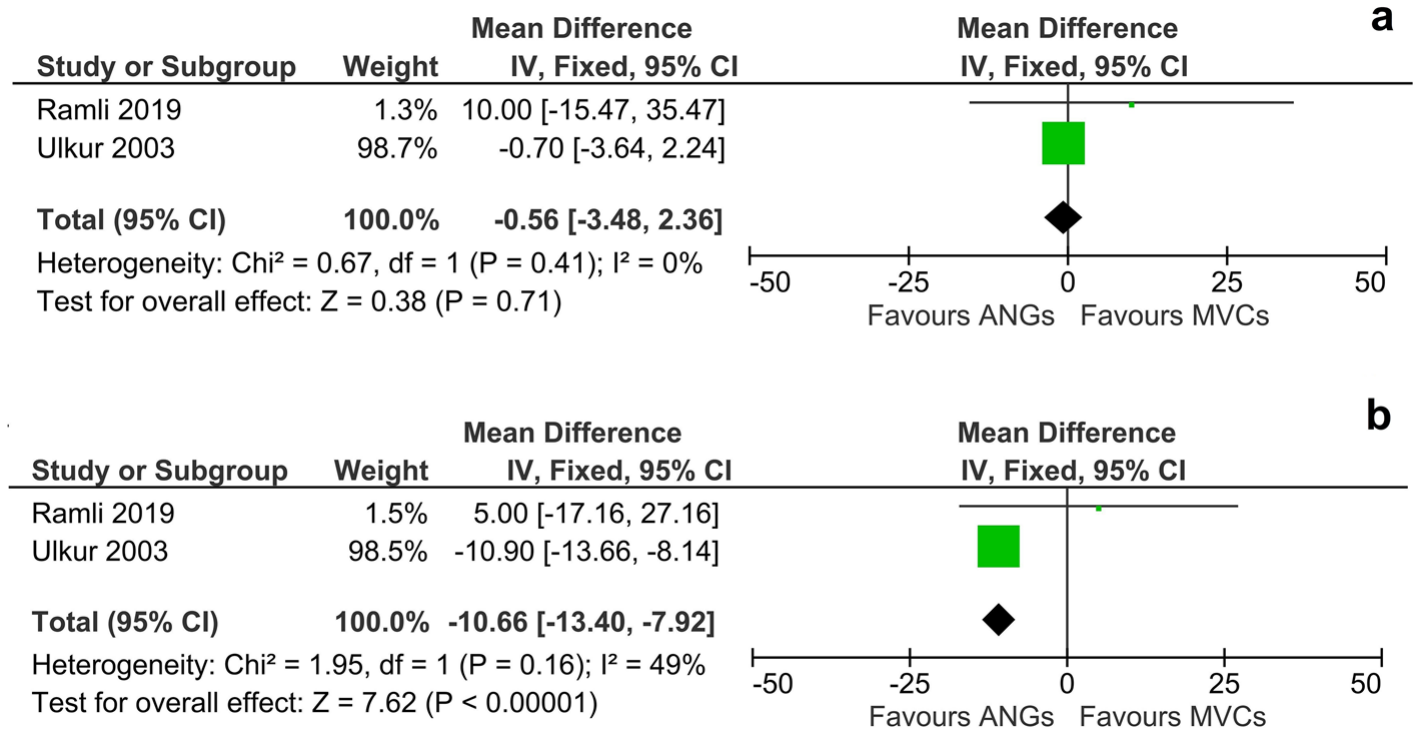
Meta-analysis of walking track performances (preclinical). Walking track performance at either 2 weeks (**a**) or 4 weeks (**b**) after sciatic nerve surgery was compared between rats who underwent nerve reconstruction either by means of an ANG or MVC. While the overall effect of the respective reconstruction method was not significant at WPO2, the meta-analysis revealed a significant effect (p < 0.01) in favor of ANGs at WPO4. While functional recovery was assessed by walking track analysis until WPO12 and WPO28, respectively, Ramli et al. excluded all animal of the MVC group starting from WPO6 due to severe autotomy of the affected limb. Therefore no meta-analysis of walking track analysis was performed at time points later than WPO4. Note that while no heterogeneity (I^2^ = 0%) was detectable at WPO2, a moderate amount of heterogeneity (I^2^ = 49%) was observable at WPO4.

Functional recovery was not significantly different following autologous nerve grafting or bridging with MVCs at WPO2 (DM, 95% CI, fixed effect: −0.56 [−3.48 to 2.36]d, I^2^ = 0%, 45 animals) (Fig. 3a). At WPO4 however, animals in the ANG group performed highly significantly better (p < 0.01) than animals in the MVC group (DM, 95% CI, fixed effect: −10.66 [−13.40 to 7.92], I^2^ = 49%, 45 animals) (Fig. 3b). Since in one study all animals in the MVC group had to be excluded from further analysis due to severe autotomy from WPO6 onwards, no later time points were included in the meta-analysis.

**Figure 4:**
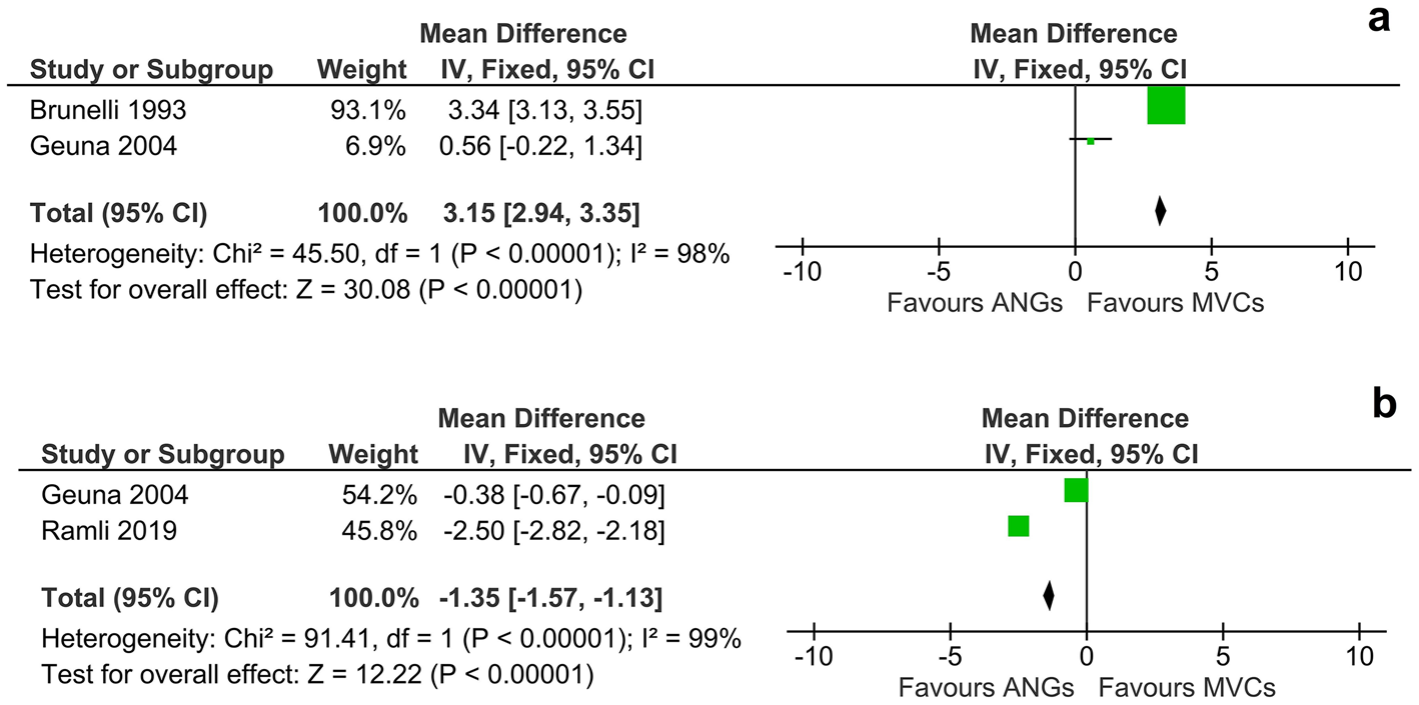
Meta-analysis of the number of axons in the distal nerve stump (**a**) and the size of the myelinated fibers (**b**) within the grafted ANG or MVC at twelve weeks following sciatic nerve reconstruction (preclinical). Analysis of axon numbers (**a**) at WPO12 revealed a statistically significant (p < 0.01) effect of nerve reconstruction by means of MVCs compared to ANGs. There was high heterogeneity (I^2^ = 98%) among the evaluated studies. Note that the scale of the Forrest-plot was adapted to 1000 axons/mm^2^ as the software allows a maximum scale of 1000. Analysis of the size of myelinated fibers (**b**) at WPO12 showed a significant effect (p < 0.01) in favor of nerve repair by means of ANGs. We observed a high heterogeneity of I^2^ = 99%.

In regard to the number of axons in the distal nerve stump at WPO12 following bridging of a 10 mm sciatic nerve defect, use of MVCs resulted in significantly (p < 0.01) higher numbers of axons than reconstruction with an ANG (DM, 95% CI, fixed effect: 3.15 [2.94 to 3.35], I^2^ = 98%, 7 animals) (Fig. 4a). In contrast to these superior results observed in the MVC group, the size of myelinated fibers was significantly (p < 0.01) higher after nerve reconstruction by means of an ANG at WPO12 (DM, 95% CI, fixed effect: −1.35 [−1.57 to −1.13], I^2^ = 99%, 7 animals) (Fig. 4b).

#### Clinical studies

**Figure 5:**
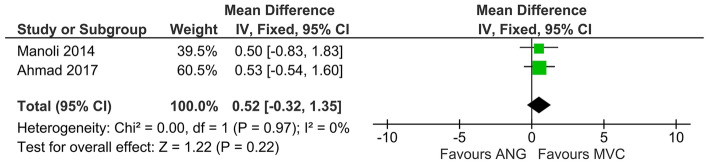
Meta-analysis of static two-point discrimination (clinical). There was no significant effect (p = 0.22) of the reconstructive method on static two-point discrimination detectable. No heterogeneity among the analyzed studies was detectable (I^2^ = 0%). Note that while the estimated effects of interventions for both studies were only slightly different (0.53 vs 0.50), Ahmad’s study was given more weight and therefore more influence on the average effect of interventions due to the narrower confidence interval in comparison to Manoli’s work (2.14 vs. 2.66).

Recovery of sensory function, evaluated by means of static two-point discrimination, was not significantly different between patients which received MVCs compared to those treated with ANGs (DM, 95% CI, fixed effect: 0.52 [−0.32 to 1.35], I^2^ = 0%, 49 cases in 46 patients) (Fig. 5). However, there was a slight trend towards better sensory function in the MVC group.

## Discussion

Reconstruction of nerve defects in which tension-free neurorrhaphy is impossible requires interposition of an adequate guiding structure to facilitate nerve regeneration. Use of ANGs demands careful consideration, regarding their limited availability and donor site morbidity. An ideal nerve conduit should be abundantly available throughout the body, with high biocompatibility and -degradability in addition to optimum biomechanical properties. The nerve within the conduit should be optimally protected from surrounding scar tissue and inflammatory cells, while its regeneration is maximally supported^7,26^. MVCs were proposed almost three decades ago by Brunelli, who suggested that their characteristics match most of the aforementioned requirements for an ideal nerve conduit^11^.

Our review and meta-analysis revealed marked differences between clinical and preclinical data in regard to the reported efficacy of MVCs and ANGs for peripheral nerve reconstruction. While the results of the included preclinical studies were mixed, clinical investigations reported non-inferiority of MVCs to ANGs. Due to strong heterogeneity in study methodology, only a limited number of studies’ data (n = 6) could be included in our quantitative synthesis, although 10 studies were eligible for inclusion in our qualitative synthesis.

Preclinical studies reported diverging results with especially recent work pointing towards inferiority of MVCs compared to ANGs. In this context, some important methodological aspects of the included studies need to be addressed. Early preclinical studies, which affirmed the high potential of MVCs, featured either a very limited number of animals, e.g. n = 3^11,25^ or reported only partial results of the respective histological and functional assessment^11,21^. Studies which were published more recently used a broader array of evaluation methods and group size was at least n = 8^17,18^. Additionally, superiority of MVCs in earlier studies was almost exclusively related to higher axon numbers in the distal nerve stump and no functional outcome was reported. In contrast, the majority of studies which assessed functional outcome reported inferior results in the MVC groups. Other authors have emphasized a discrepancy between axon numbers and the degree of functional recovery following experimental peripheral nerve injury and regeneration^27^. Since nerve regeneration consists of (1) axonal regrowth (2) end-organ reinnervation and (3) functional recovery^28^ the overall outcome should not be judged based on results of only one of these three phases. As comprehensive analyses of functional recovery were primarily published in recent works, there is a growing body of literature indicating inferiority of MVCs to ANGs in preclinical murine models.

Clinical evaluations of MVCs were performed in a number of studies without an ANG control group^10,29–31^. We found only two studies that compared functional outcome to ANGs, both reporting non-inferiority of MVCs for reconstruction of upper extremity nerve defects.

The underlying reasons for these observed discrepancies between preclinical and clinical results remain to be elucidated. In our opinion, three major points must be addressed.

First, the way the grafts are prepared and coaptated to the nerve stumps is of crucial relevance regarding the outcome of nerve reconstruction. Tension-free nerve repair has been emphasized by Millesi as paramount prerequisite for optimal nerve regeneration^32^ due to the devastating consequences of tension on intraneural perfusion^33^. However, there might be differences regarding graft coaptation between preclinical and clinical studies. In the clinical situation, the patient’s nerve has already been severed, therefore a segmental damage cannot be bridged with the original nerve. Thus, if an MCV or ANG is placed between the nerve stumps, the surgeon chooses the respective transplant’s length to guarantee tension-free coaptation. In preclinical studies however, the nerve gap is created by the experimenter and needs to be of standardized length to guarantee comparability of experimental results. In consequence, for ANG repair the gap is usually bridged with the original nerve in retrograde fashion. However, given the tendency of the nerve stumps to retract the nerve gap increases by < 3 mm^34^. Therefore, tension-free coaptation might be more difficult. In addition, we assume that most experimenters prepare MVCs of the same length as the ANGs, possibly hampering tension-free repair. This in turn might complicate pulling the nerve stumps inside the MVC, a crucial aspect of nerve repair by means of this approach^20^. If the nerve is not pulled inside the MVC regenerating axons might get trapped outside, hindering regeneration^12^.

Second, clinical studies of peripheral nerve repair most commonly feature injuries of digital nerves, which are relatively small and monofascicular^35^. In opposite, mixed (motor and sensory) multifascicular nerves are most commonly studied in preclinical studies. Misdirection of axons, which significantly impairs recovery of target organ function^36^, occurs more likely when a gap of a mixed nerve is bridged with an MVC, which lacks the fascicular pattern of the original nerve. This is supported by a study reporting non-inferiority of MVCs compared to primary neurorrhaphy in rats with distal facial nerve injury. Since the nerve is mostly comprised of muscular fibers at this level, axonal misdirection might be less devastating for functional recovery^37^.

Third, the process of neuroregeneration, despite striking similarities, is markedly different in certain aspects between rodents and humans^38,39^. As prerequisite for successful nerve regeneration, cellular debris and other potential obstacles for regrowing axons must be removed following Wallerian degeneration^40,41^. Next, a fibrin matrix forms between the nerve stumps, acting as a guiding scaffold for regenerating axons^42^. While the fibrin cable is degraded within two weeks in humans, it lasts around 4 weeks in rats. This defines the maximum length of axonal regeneration without interposition of a guidance structure, e.g. the “critical size defect”, which is ~ 1.5 cm in rats and ~ 4 cm in humans^38^. It was established that longitudinally oriented fibers, e.g. collagen or muscle, can support axonal regrowth and functional recovery^11,43,44^. However, nerve regeneration is hindered if these guidance structures are degraded before regenerating axons reach them. On the other hand, the fibers must be fully degraded before nerve regeneration is finished, otherwise complete recovery will be impeded^45^. The muscle fibers inside an MVC^46^ are degraded by proteases while simultaneous axon regrowth through the fibrin cable occurs. It was shown that an MVC collapses as soon as all muscle fibers within have been degraded, which decidedly hinders axonal regeneration^17^. As shown for mice, rodents possess about 1.4 × the number of proteases and have a markedly increased proteome turnover in comparison to humans^47^. This is thought to be related to their shorter lifespan with their body optimized to quickly regain optimal integrity and function rather than maintaining the body over a long time^48^. We therefore hypothesize, that muscle fibers inside MVCs are degraded markedly faster in rodents than in humans, contributing to the inferior results of MVC nerve repair in murine models. As human proteome turnover is slower, muscle fibers inside the MVC persist longer, allowing more time for axons to transverse the MVC before it collapses.

These profound differences impair clinical translation of results in rodent studies of peripheral nerve repair, especially in regard to critical size defects. However, critical size nerve defects represent a common clinical problem with a high need for adequate treatment options^38,39^. Out of the eight preclinical studies included in this work, three featured a critical size nerve defect. Two of these studies were published within the last 3 years and included at least two assessments for functional recovery each. Notably, all three studies reported inferiority of MVCs to ANGs for bridging long nerve defects in rodents. Clinical results of MVCs vs ANGs after reconstruction of peripheral nerve defects > 3 cm were only reported by Manoli et al.^12^. The authors reconstructed four 4–6 cm gaps, two each with an ANG or an MVC. Interestingly, they reported a good and a poor result each for both methods. Due to the limited numbers of patients, no statistical analysis could be performed between the group of patients with 4–6 cm defects and the group with smaller nerve gaps. Since long defects of 6 cm and more are often associated with markedly worse or even no functional recovery^34^, it could be assumed that the overall results, e.g. non-inferiority of MVCs to ANGs, were at least partially influenced by the length of the nerve gaps in this study. Other authors published good results after repair of 4–6 cm long nerve gaps with MVCs, but their study lacked an ANG group^10^. Therefore, no concise conclusion is possible in regard to the feasibility of MVCs vs ANGs for reconstruction of long nerve defects in human patients ^49^.

Although promising results for MVCs were obtained in a small number of clinical studies, there is evident need for larger, clinical trials to gather more data. Based on the results of this work we see a strong need to investigate MVCs in the setting of a critical size nerve defect in a non-rodent model. The disadvantages of rodent models, although cost-efficient and commonly used, have been pointed out by us. Large mammalians models such as sheep or pigs come with considerably higher costs as a disadvantage ^50^. However, the results of these animal models are more easily transferable to human patients ^51^. Therefore, we advise evaluation of MVCs in large animal models before further clinical studies involving mixed motor nerves or nerve gaps of critical size should be intended.

## Conclusion

Our results underpin that preclinical studies in rodents are not able to adequately prognose the feasibility of MVCs for reconstruction of critical-size defects in humans. This can be explained by differences regarding the experimental settings, and most important, profound inter-species differences in neurobiology. Additionally, data from clinical trials, in particular involving defects of mixed nerves, are still very sparse. Although large prospective human studies to answer these questions will be inevitable, we strongly advise for large animal studies beforehand. Our work emphasizes two main problems of peripheral nerve research: limited translatability of results from rodents to humans and profound differences regarding study methodology. While MVCs tackle several main problems of peripheral nerve repair by means of an abundantly available, autologous material with high biocompatibility, their promising features have not yet been explored in enough detail to support their unfettered use for nerve repair in human patients.

## Methods

According to the PRISMA statement^52^ and in adherence with the recommendations found in the literature^53^, a systematic review and meta-analysis was conducted to investigate the use of MVCs as alternative to ANGs for reconstruction of segmental peripheral nerve defects. Both preclinical and clinical studies were featured in the data synthesis.

### Search strategy, eligibility, and inclusion criteria

In accordance with the PRISMA guidelines and exemplary published work^53^, a systematic search strategy (Supplementary Table 1) was designed in August 2020 for the database PubMed and Web of Science core collection. The latest literature research was performed on December 1st, 2020. All identified studies in this research were then screened for general eligibility and inclusion using a predefined list of inclusion criteria both for the preclinical as well as the clinical studies (Table 1). All experimental animal studies in rats which compared MVCs against the gold standard of ANGs were hypothetically eligible for inclusion into the analysis of preclinical data. Regarding the analysis of clinical data, all clinical studies investigating the use of MVCs versus ANGs to reconstruct segmental nerve defects in humans were theoretically deemed eligible. Studies which were identified through literature research but did not meet the inclusion criteria were excluded from the analysis (Supplementary Table 2 + Supplementary Table 3).

### Selection of studies and extraction of data

Following the initial literature research, the identified studies’ abstracts and titles were screened to identify all studies potentially eligible for inclusion. All studies meeting these criteria were obtained in full text. There studies were then assessed thoroughly for their eligibility for inclusion and their reference lists were used to search for additional relevant publications. In the next step, all relevant data was extracted (Tables 2, 3) and prepared for analysis. Since the degree of functional recovery is the most important criterion to determine the feasibility and effects of a reconstructive technique in peripheral nerve repair^54^, we chose recovery of voluntary motor function as the primary outcome parameter for the preclinical studies.

**Table 2:** Extracted details of the included preclinical studies.

Study	Nerve	Defect size	Surgical methods	Included animals	Observation period	Outcomes
Brunelli ^11^	Sciatic	(A) 10 mm (B) 20 mm	(1) ANG (2) Muscle alone (3) Empty vein graft (4) MVC	n = 24	12 weeks	Functional examination (not further specified), number of axons
Geuna ^25^	Sciatic	10 mm	(1) Control (2) MVC (3) ANG	n = 12	12 weeks	Total number, mean density and size of myelinated fibers
Geuna ^21^	Median	10 mm	(1) End-to-side repair using the ulnar nerve (2) ANG (3) MVC (4) Y-shaped MVC on median nerve (5) Y-shaped MVC on ulnar nerve	n = 72	10 months	Functional examination (grasping test), histological analysis
Papalia ^24^	Sciatic	10 mm	(1) Epigastric vein filled with adipose tissue (2) ANG (3) MVC	n = 20	6 months	Functional examination (grasping test), total number, diameter of myelinated fibers and myelin thickness after 6 months
Ramli ^20^	Sciatic	15 mm	(1) ANG (2) No treatment (3) MVC with neural transdifferentiated MSCs (4) unseeded MVC (5) polyglycolic acid nerve conduit	n = 15	12 weeks	Sensory function (pinch test) and motor function assessment (walking tracks), nerve conduction velocity, fiber diameter, axon diameter and myelin thickness
Stössel ^18^	Median	7 mm	(1) MVC (2) ANG	n = 16	8 weeks and 12 weeks	Functional examination (grasping test, staircase test), nerve conduction velocity every 4 weeks, diameter of myelinated fibers and axons, myelin thickness
Stössel ^17^	Sciatic	15 mm	(A) immediate repair B) repair postponed for 45 days (1) regular chitosan (2) novel chitosan (3) ANG (4) MVC	n = 56	120 days (acute repair), 150 days (delayed)	Transcutaneous electrodiagnostic, muscle weight ratios, total nerve fiber number, nerve fiber density, diameter of axons and fibers and myelin thickness
Ülkür ^22^	Sciatic	20 mm	(1) ANT (2) vein only (3) MVC	n = 30	28 weeks	Functional examination (walking track) in week 2, 4, 8, 12, 20, and 28, number of myelinated axons and mean axonal diameter

**Table 3:** Extracted details of the included clinical studies. *: Note that the number of cases was higher than the number of patients because three patients in the MVC group each underwent reconstruction of two digital nerve defects with MVCs.

Study	Nerves	Defect size	Surgical methods	Included patients	Age range	Observation period	Outcomes
Ahmad ^16^	Median Ulnar	< 30 mm	(1) ANG using the sural nerve (2) MVC	n = 18 (1) n = 9 (2) n = 9	1) 27.22 ± 5.35 2) 26.80 ± 5.50	not reported	Static two-point discrimination, muscle power
Manoli ^12^	Digital	10 – 60 mm	(1) ANG using the medial antebrachial cutaneous nerve (2) MVC	n = 28* Cases: (1) n = 14 (2) n = 17	11 – 72 years	12 – 58 months	Semmes–Weinstein monofilaments, static + moving two-point discrimination

Given the predominance of the rat sciatic nerve model in preclinical peripheral nerve research^55^, calculation of the Sciatic Functional Index (SFI) or Peroneal Functional Index (PFI) by means of walking track analysis are well-established tools to determine the degree of functional recovery in this context^56^. Established by de Medinaceli et al. in 1982, these methods include dipping the rat’s hind paws in ink to be subsequently recorded and measured. While values around zero indicate normal function, an SFI of around -100 equals complete loss of function^57^. Sensory recovery can be evaluated by means of the “Pinch Test”, in which toothed forceps are used to nip the toes of the respective paw.

Besides the rat sciatic nerve model, the median nerve model is another frequently used model in preclinical research^58^. Voluntary motor function in this model is most commonly assessed by means of the grasping test. While being held at its tail, the rat grasps a bar connected to a scale to measure and record the maximum force exercisable by the finger flexor muscles. As these are predominantly innervated by the median nerve in rats, this method allows to determine the degree of functional recovery after median nerve reconstruction. The staircase test is an alternative method to assess motor function following median nerve injury and reconstruction. It evaluates the fine motor skills of the forelimb while the examined rat retrieves spheroid-shaped pellets of food from a staircase apparatus with restricted latitude^18^. As secondary outcome, electrophysiological parameters such as the nerve conduction velocity (NCV) or the compound muscle action potential (CMAP) of the respective target muscles were evaluated. Muscle weights of target muscles were chosen as complementary outcome metrics. Additionally, histological and histomorphometric parameters, e. g. the diameter of myelinated axons, the myelin sheath thickness, g-ratio and the density or number of myelinated nerve fibers distal to the lesion site or inside the MVC and ANG were assessed. Regarding the clinical studies included in our synthesis, recovery of sensory function as assessed by static two-point discrimination was chosen as primary outcome parameter. Additional outcome metrics were moving two-point discrimination, the Semmes–Weinstein-monofilament test which is used to assess cutaneus sensory thresholds by applying nylon filaments of different bendability to the respective skin area, and the Medical Research Council scale^59^ for muscle strength. The methodological quality of all included studies was assessed and the risk of bias was judged in accordance with the recommendations of the Cochrane Handbook for Systematic Reviews of Interventions^60^ for the following aspects: random sequence generation and allocation concealment (selection bias), blinding of participants and personnel (performance bias), blinding of outcome assessment (detection bias), incomplete outcome data (attrition bias), selective reporting (reporting bias) and other bias, e.g. suspected insufficient statistical power or methods or use of non-standardized or strongly modified experimental methods.

### Statistical analysis, qualitative and quantitative synthesis of data

We performed the meta-analysis and created the respective figures using Cochrane statistical software Review Manager 5.3^61^ (The Cochrane Collaboration, Copenhagen, the Nordic Cochrane Centre). Preclinical studies were clustered into two groups, either featuring the sciatic nerve or median nerve model. Further statistical subgroup analysis was based on this sorting. However due to a strong heterogeneity regarding the assessed outcomes, length of the observation period and surgical methodology, a meta-analysis could only be performed on the sciatic nerve model group. We used the inverse variance (IV) method in a fixed effect analysis-model and expressed the results as difference in means (DM) for continuous outcomes with 95% confidence interval (CI). Since the Review Manager requires the standard deviation for each outcome, but some studies reported the standard error of the mean (SEM) instead, the Standard Deviation was calculated by multiplying the square root of the sample sizes with the SEM. The weight of the estimated intervention effect of each individual study was then determined based on the width of the confidence intervals of the respective study. In conclusion, studies with narrower confidence intervals had a higher impact on the overall average effect of interventions for the respective reconstructive approach (MVC or ANG respectively)^62^. Heterogeneity among studies was assessed by means of the Chi^2^ and I^2^ test^63^.

## Supplementary Information

Supplementary Information.

## Data Availability

The original contributions presented in the study are included in the article, further inquiries can be directed to the corresponding author.
