# Supplementary material for: A systematic review and meta-analysis of studies comparing muscle-in-vein conduits with autologous nerve grafts for nerve reconstruction
Source: Sci Rep. 2021 Jun 3;11:11691. doi: 10.1038/s41598-021-90956-3 (PMC8175734; doi:10.1038/s41598-021-90956-3)
Supplement: Supplementary file 1 — Supplementary Information. [file 41598_2021_90956_MOESM1_ESM.pdf]

A systematic review and meta-analysis of studies comparing muscle-in-vein conduits with autologous nerve grafts for nerve reconstruction

Johannes C. Heinzel[1,2,3*], Mai Quyen-Nguyen[2,3], Laura Kefalianakis[1], Cosima Prahm[1], Adrien Daigeler[1], David Hercher[2,3], Jonas Kolbenschlag[1]

[1] Department of Hand-, Plastic, Reconstructive and Burn Surgery, BG Klinik Tuebingen, Eberhard Karls University, Schnarrenbergstraße 95, Tuebingen, Germany

[2] Ludwig Boltzmann Institute for Experimental and Clinical Traumatology, Donaueschingenstraße 13, Vienna, Austria

[3] Austrian Cluster for Tissue Regeneration

*** Correspondence:**
Johannes C. Heinzel
hannes.heinzel@gmx.de

Supplementary Material

Supplementary Table 1 – Search terms and results of the literature research according to the PRISMA guidelines.

Search term(s)	Results from PubMed	Results from Web of Science
muscle-in-vein AND conduit	6	10
muscle-in-vein AND graft	8	13
muscle-vein AND conduit	13	15
muscle-vein AND graft	22	26
muscle-vein AND nerve	26	48
muscle-in-vein AND nerve	10	14
“muscle and vein conduit”	4	1
“muscle and vein conduits”	5	1
“muscle and vein graft”	1	0
“muscle and vein grafts”	3	0
“muscle-stuffed vein”	3	6
“vein filled with muscle”	5	6
“vein” AND "muscle graft*"	22	48
“vein conduit*” AND “nerve”	55	80
**Total**	183	268

Supplementary Table 2 – Excluded preclinical studies.

Study	Reason for exclusion
Battiston [1]	No control group (ANG) featured
Couturier [2]	No control group (ANG) featured
Di Benedetto [3]	No control group (ANG) featured
Fernandes [4]	Full-text not available in English
Fornaro [5]	Reported outcome measures not relevant for synthesis
Geuna [6]	Reported outcome measures not relevant for synthesis
Geuna [7]	Full-text not available
Geuna [8]	Reported outcome measures not relevant for synthesis

Geuna [9]	Reported outcome measures not relevant for synthesis
Li [10]	No control group (ANG) featured
Mohammadi [11]	No control group (ANG) featured
Nicolino [12]	No control group (ANG) featured
Nijhuis [13]	Non-standardized way to prepare the MVCs
Pagnotta [14]	Reported outcome measures not relevant for synthesis
Raimondo [15]	Reported outcome measures not relevant for synthesis
Tos [16]	Reported outcome measures not relevant for synthesis
Tos [17]	Reported outcome measures not relevant for synthesis
Tos [18]	Reported outcome measures not relevant for synthesis
Tuncel [19]	Non-standardized way to prepare the MVCs
Manthou [20]	No control group (ANG) featured
Jemnoschi-Hreniuc ([21]	Non-standardized way to prepare the MVCs

Supplementary Table 3 – Excluded clinical studies.

Study	Reason for exclusion
Battiston [22]	No control group (ANG) featured
Battiston [23]	No control group (ANG) featured
Marcoccio [24]	No control group (ANG) featured
Schiefer [25]	No control group (ANG) featured

1. Battiston, B., Tos, P., Geuna, S., Giacobini-Robecchi, M.G. & Guglielmone, R. Nerve repair by means of vein filled with muscle grafts. II. Morphological analysis of regeneration. *Microsurgery* **20**, 37-41 (2000).
2. Couturier, C.A., Dauge, M.C., Henin, D., Alnot, J.Y. & Masmejean, E.H. Nerve repair using a composite graft of vein and denatured skeletal muscle: morphologic analysis. *J Reconstr Microsurg* **18**, 681-688 (2002).
3. Di Benedetto, G., *et al.* Nerve regeneration through a combined autologous conduit (vein plus acellular muscle grafts). *Biomaterials* **19**, 173-181 (1998).
4. Fernandes, M., *et al.* Comparative study between autogenous graft and muscular graft covered with autogenous vein tube in Wistar rats' tibial nerves using the Fluoro-Gold® as a neuronal marker. *Acta Ortopédica Brasileira* **15**, 97-100 (2006).
5. Fornaro, M., Tos, P., Geuna, S., Giacobini-Robecchi, M.G. & Battiston, B. Confocal imaging of Schwann-cell migration along muscle-vein combined grafts used to bridge nerve defects in the rat. *Microsurgery* **21**, 153-155 (2001).
6. Geuna, S., Tos, P., Battiston, B., Guglielmone, R. & Giacobini-Robecchi, M.G. Morphological analysis of peripheral nerve regenerated by means of vein grafts filled with fresh skeletal muscle. *Anat Embryol (Berl)* **201**, 475-482 (2000).
7. Geuna, S., Tos, P., Battiston, B., Guglielmone, R. & Giacobini-Robecchi, M.G. A stereological study of long-term regeneration of rat severed sciatic nerve repaired by means of muscle-vein-combined grafts. *Ital J Anat Embryol* **105**, 65-73 (2000).
8. Geuna, S., *et al.* Schwann-cell proliferation in muscle-vein combined conduits for bridging rat sciatic nerve defects. *J Reconstr Microsurg* **19**, 119-123; discussion 124 (2003).
9. Geuna, S., *et al.* Nerve regeneration along bioengineered scaffolds. *Microsurgery* **27**, 429-438 (2007).

10. Li, Y.L., Gao, Z.Q., Wang, Z.L., Liu, Y.G. & Zhang, Q.H. Autogenous inside-out versus standard vein and skeletal muscle-combined grafting for facial nerve repair. *Neural Regeneration Research* **5**, 282-286 (2010).

11. Mohammadi, J., Delaviz, H., Mohammadi, B., Delaviz, H. & Rad, P. Comparison of repair of peripheral nerve transection in predegenerated muscle with and without a vein graft. *BMC Neurol* **16**, 237 (2016).

12. Nicolino, S., *et al.* Expression of alpha2a-2b neuregulin-1 is associated with early peripheral nerve repair along muscle-enriched tubes. *Neuroreport* **14**, 1541-1545 (2003).

13. Nijhuis, T.H., *et al.* Natural conduits for bridging a 15-mm nerve defect: comparison of the vein supported by muscle and bone marrow stromal cells with a nerve autograft. *J Plast Reconstr Aesthet Surg* **66**, 251-259 (2013).

14. Pagnotta, A., *et al.* Neurotrophins and their receptors in early axonal regeneration along muscle-vein-combined grafts. *Microsurgery* **22**, 300-303 (2002).

15. Raimondo, S., *et al.* Schwann cell behavior after nerve repair by means of tissue-engineered muscle-vein combined guides. *J Comp Neurol* **489**, 249-259 (2005).

16. Tos, P., *et al.* Tissue specificity in rat peripheral nerve regeneration through combined skeletal muscle and vein conduit grafts. *Microsurgery* **20**, 65-71 (2000).

17. Tos, P., *et al.* Use of muscle-vein-combined Y-chambers for repair of multiple nerve lesions: experimental results. *Microsurgery* **24**, 459-464 (2004).

18. Tos, P., *et al.* Comparison of fresh and predegenerated muscle-vein-combined guides for the repair of rat median nerve. *Microsurgery* **27**, 48-55 (2007).

19. Tuncel, U., *et al.* The Effect of Autologous Fat Graft with Different Surgical Repair Methods on Nerve Regeneration in a Rat Sciatic Nerve Defect Model. *Plast Reconstr Surg* **136**, 1181-1191 (2015).

20. Manthou, M.E., *et al.* Facial nerve repair by muscle-vein conduit in rats: functional recovery and muscle reinnervation. *Tissue Eng Part A* (2020).

21. Jemnoschi-Hreniuc, I., *et al.* Prolonging Nerve Grafts Using Chemical Extracted Muscle-in-vein with Vein Window Method Chemical acellular nerve grafts. *Revista de Chimie -Bucharest- Original Edition-* **December**(2017).

22. Battiston, B., Tos, P., Cushway, T.R. & Geuna, S. Nerve repair by means of vein filled with muscle grafts I. Clinical results. *Microsurgery* **20**, 32-36 (2000).

23. Battiston, B., Tos, P., Conforti, L.G. & Geuna, S. Alternative techniques for peripheral nerve repair: conduits and end-to-side neurorrhaphy. *Acta Neurochir Suppl* **100**, 43-50 (2007).

24. Marcoccio, I. & Vigasio, A. Muscle-in-vein nerve guide for secondary reconstruction in digital nerve lesions. *J Hand Surg Am* **35**, 1418-1426 (2010).

25. Schiefer, J.L., *et al.* Comparison of short- with long-term regeneration results after digital nerve reconstruction with muscle-in-vein conduits. *Neural Regen Res* **10**, 1674-1677 (2015).
